# The Emergence and Zoonotic Transmission of H10Nx Avian Influenza Virus Infections

**DOI:** 10.1128/mBio.01785-21

**Published:** 2021-09-07

**Authors:** Holly Everest, Elizabeth Billington, Rebecca Daines, Alice Burman, Munir Iqbal

**Affiliations:** a The Pirbright Institute, Pirbright, Woking, Surrey, United Kingdom; b Nuffield Department of Medicine, University of Oxford, Headington, Oxford, United Kingdom; c The Roslin Institute, The University of Edinburgh, Midlothian, United Kingdom; d Pathobiology and Population Sciences, Royal Veterinary College, Hatfield, Hertfordshire, United Kingdom; Albert Einstein College of Medicine; Albert Einstein College of Medicine

**Keywords:** avian influenza, avian viruses, H10Nx, pandemic, poultry, reassortment, zoonotic

## Abstract

Avian influenza viruses pose a continuous threat to both poultry and human health, with significant economic impact. The ability of viruses to reassort and jump the species barrier into mammalian hosts generates a constant pandemic threat. H10Nx avian viruses have been shown to replicate in mammalian species without prior adaptation and have caused significant human infection and fatalities. They are able to rapidly reassort with circulating poultry strains and go undetected due to their low pathogenicity in chickens. Novel detections of both human reassortant strains and increasing endemicity of H10Nx poultry infections highlight the increasing need for heightened surveillance and greater understanding of the distribution, tropism, and infection capabilities of these viruses. In this minireview, we highlight the gap in the current understanding of this subtype and its prevalence across a vast range of host species and geographical locations.

## INTRODUCTION

Influenza A viruses (IAV), which include avian influenza viruses (AIV), are members of the family *Orthomyxoviridae* and are negative-sense, single-stranded viruses containing a segmented RNA genome. Each genome consists of eight viral RNA (vRNA) segments enveloped in a host-derived lipid membrane with two surface glycoproteins: hemagglutinin (HA) and neuraminidase (NA). Variations in antigenicity and phylogenetics of the HA and NA proteins allow their characterization into subtypes, designated H(x)N(y). Eighteen HA subtypes and 11 NA subtypes have so far been identified. Individual isolates are named according to the following pattern: antigenic type (A, B, C, or D), host of origin, geographical location, strain number, and year of isolation (1). This is then succeeded by H(x)N(y). Where the host of isolation is human, the host of origin is excluded from the strain name.

The primary natural host reservoir of IAVs is wild aquatic birds (predominately *Anseriformes*) (2, 3) with the exception of H17 and H18 subtype viruses isolated only from bats (4, 5). AIVs may also cause sporadic incursions in domestic poultry, humans, and other mammalian species (6, 7). The recent identification of a novel reassortant H10N3 virus in humans has reignited concerns regarding the pandemic potential of the H10 influenza subtype (8). H10 viruses with nine NA subtypes have been isolated from various hosts. However, all have been wild bird derived and evolved to infect both terrestrial poultry and mammalian species.

AIVs can be classified into two pathotypes both by molecular signature within their HA segment and as a result of clinical disease in chickens, referred to as low-pathogenicity avian influenza virus (LPAIV) and high-pathogenicity avian influenza virus (HPAIV) (9). Specifically, a virus is considered an HPAIV if it has an intravenous pathogenicity index (IVPI) score in 6-week-old chickens greater than 1.2 or causes at least 75% mortality in 4- to 8-week-old chickens when birds are infected intravenously (10, 11). The virus is also considered an HPAIV if there is a polybasic cleavage site in the HA segment and endogenous furin-like proteases activate the HA at polybasic cleavage sites to facilitate a systemic, and often fatal, infection. Only subtypes H5 and H7 have displayed this phenotype in natural isolates. However, an H10 isolate (A/Mandarin duck/Singapore/805/F-72/7/1993) was found to be highly pathogenic via *in vivo* testing procedures despite H10 AIVs not usually being associated with high pathogenicity (12). A point to note is that the virus was atypical of HPAIVs in that it was not pathogenic when administered intranasally and did not possess a polybasic cleavage site. It also did not replicate in the brains of chickens after intravenous inoculation. An earlier H10 isolate (A/Turkey/England/384/79) suggested that it also had similarly pathogenicity and that the pathogenicity may be attributed to replication in the kidney (12). Absence of the polybasic cleavage site usually classifies the virus as being LPAIV (13, 14), but the presence of di- or tribasic cleavage sites can lead to enhanced pathogenicity (15). LPAIV typically causes milder clinical disease in poultry, often associated with high morbidity (>50%) and low mortality (<5%) (16, 17). However, in some cases of LPAIV infection, mortality can increase, depending on the host susceptibility as well as instances of concurrent or secondary infection with other diseases (16, 18, 19).

While there have been cases in avian species of infection with all nine H10 subtypes, only H10N8 (20), H10N7, and more recently H10N3 (8) have been detected in humans. Zoonotic transmission events are fairly infrequent, and the resulting human infections have raised concerns over virus origin, evolution, and potential human-human transmission. The emergence and prevalence of H10 viruses in both chickens and the occurrence of human infections provide direct evidence of the threat from currently circulating H10Nx infections.

## HISTORY AND EMERGENCE OF H10Nx VIRUSES

### History of H10Nx isolation and detection.

The H10 subtype has been isolated from both terrestrial avian species and wild and domestic aquatic birds globally. The first detection of H10 subtype viruses was in chickens in Germany in 1949 [A/Chicken/Germany/N/1949 (H10N7)]. The first detection of H10Nx infection outside Europe was in North America, specifically in Canada, in 1953 [A/Duck/Manitoba/1953 (H10N7)].

### Species and geographical distribution of H10Nx viruses.

H10Nx infections are enzootic in both poultry and wild bird populations globally. They also continuously circulate in live-bird markets (LBMs) in Asia. Surveillance studies have indicated that terrestrial poultry were the root cause of H10Nx zoonotic transmission events in China beginning in 2012 (20–22). H10Nx viruses have a broad host range and are capable of reassorting with increased fitness for mammalian species (23).

### Global surveillance of H10Nx viruses.

While surveillance for notifiable AIV subtypes (H5 and H7) is common practice, for neglected subtypes, including H10Nx, surveillance is typically poor. Surveillance of AIV is often limited to subtype prevalence in bird populations based on sequences of HA and NA glycoproteins, but some studies are able to investigate internal genes to examine viral evolution and possible virulence factors (24–26). Internal viral genes are sequenced to determine presence of zoonotic risk mutations or virulence markers of specific segments (27) or to track the reassortment of internal genes (24, 25, 28), which is of particular importance for H10Nx infections, as they have been shown to frequently reassort with other circulating subtypes. In many parts of the world, surveillance is conducted in response to disease outbreaks rather than being a long-term project (25), which can make it hard to elucidate long-term trends. Long-term general AIV surveillance has shown that migratory birds have introduced H10 viruses into terrestrial poultry populations, which have caused sustained outbreaks, resulting in identification of numerous reassortant viruses (29) (Table 1).

**TABLE 1:** All H10 HA sequences currently available on www.fludb.org, with the corresponding years of detection and sampling sources^*a*^

Country	Period	Subtype	Species	No. of strains	Total
Argentina	2011	H10N7	Silver teal, yellow-billed pintail	2	2
Australia	2010	H10N7	Human	2	3
	1972	H10N8	Shearwater	1	
Bangladesh	2019	H10N3	Duck (unspecified)	2	11
	2019	H10N4	Duck (unspecified)	2	
	2015	H10N6	Duck (unspecified)	1	
	2009	H10N7	Chicken, duck (unspecified)	4	
	2010	H10N9	Duck (unspecified)	2	
Belgium	2018	H10N1	Mallard	1	3
	2016–2017	H10N5	Mallard	2	
Canada	1995	H10N1	Mallard	1	46
	1978–2013	H10N3	Blue-winged teal, mallard	2	
	1984–2010	H10N6	American black duck, pintail, mallard	4	
	1953–2016	H10N7	American black duck, pintail, mallard, blue-winged teal, pintail (unspecified), duck (unspecified), scaup, mallard-black duck hybrid	39	
Chile	2016	H10N7	Yellow-billed pintail	3	3
China	2015	H10N1	Duck (unspecified)	1	172
	2005–2013	H10N2	Duck (unspecified)	2	
	2005–2017	H10N3	Duck (unspecified), chicken	39	
	2003–2014	H10N5	Duck (unspecified), swine, wild bird (unspecified)	11	
	2005–2014	H10N6	Duck (unspecified), chicken	35	
	2008–2016	H10N7	Duck (unspecified), goose (unspecified), mallard, chicken	20	
	2005–2016	H10N8	Duck (unspecified), Environment, chicken, human	63	
	2013	H10N9	Chicken	1	
Egypt	2015	H10N7	Teal (unspecified)	1	1
Germany	1949	H10N7	Bird (unspecified), chicken	3	3
Georgia	2011	H10N4	Mallard	1	6
	2010–2014	H10N7	Mallard, duck (unspecified)	5	
Hong Kong	1980	H10N5	Mallard	1	6
	1979–2009	H10N9	Duck (unspecified), northern shoveler, teal (unspecified)	5	
Iceland	2015	H10N7	Black-headed gull, glaucous gull, Iceland gull	4	4
Italy	1965	H10N8	Quail	2	2
Japan	2007	H10N2	Duck (unspecified)	2	12
	2013	H10N3	Waterfowl (unspecified)	1	
	2000–2010	H10N4	Waterfowl (unspecified), duck (unspecified)	3	
	2008–2014	H10N7	Waterfowl (unspecified), duck (unspecified), mallard	5	
	2008	H10N9	Waterfowl (unspecified)	1	
Mexico	2009	H10N7	Green-winged teal	1	1
Mongolia	2015	H10N2	Mallard, duck (unspecified)	4	29
	2001–2015	H10N3	Waterfowl (unspecified), duck (unspecified), mallard	8	
	2014	H10N6	Waterfowl (unspecified), duck (unspecified)	2	
	2011–2015	H10N7	Waterfowl (unspecified), mallard	9	
	2010–2014	H10N8	Waterfowl (unspecified), duck (unspecified)	6	
Netherlands	2007	H10N1	Eurasian wigeon	2	32
	2006–2008	H10N4	Herring gull, mallard, ruddy turnstone	5	
	2006	H10N6	Mallard	2	
	2006–2015	H10N7	Mallard, mute swan	23	
Sweden	2007–2009	H10N1	Mallard	20	60
	2002	H10N2	Mallard	3	
	1984–2011	H10N4	Mallard, mink	15	
	2007	H10N5	Mallard	2	
	2002–2009	H10N6	Mallard	4	
	2002–2007	H10N7	Mallard	6	
	2002–2003	H10N8	Mallard	3	
	2007	H10N9	Mallard	7	
United Kingdom	1985	H10N4	Fowl (unspecified), mallard	2	2
USA	2006–2011	H10N1	Northern shoveler, ruddy turnstone, shorebird, mallard, common goldeneye, red knot	32	824
	1987–2018	H10N2	Green-winged teal, laughing gull, shorebird, ruddy turnstone, northern shoveler, common eider, coot, environment, greater scaup, mallard, red knot, shorebird	42	
	1984–2017	H10N3	American wigeon, blue-winged teal, cinnamon teal, common goldeneye, coot, duck (unspecified), environment, green-winged teal, lesser scaup, mallard, northern shoveler, ruddy turnstone	73	
	2000–2019	H10N4	Environment, mallard, northern pintail, red knot, ruddy turnstone, shorebird	31	
	1987–2016	H10N5	Knot (unspecified), ruddy turnstone, mallard, green-winged teal, environment, red knot, gull (unspecified), semipalmated sandpiper, laughing gull, sanderling	96	
	1989–2014	H10N6	Mallard, American green-winged teal, blue-winged teal, northern shoveler, northern pintail, long-tailed duck, red knot, ruddy turnstone, scoter	11	
	1979–2019	H10N7	American green-winged teal, black scoter, blue-winged teal, common goldeneye, common murre, duck, environment, Eurasian teal, gadwall, goose (unspecified), greater white-fronted goose, green-winged teal, gull (unspecified), herring gull, laughing gull, lesser scaup, mallard, northern pintail, northern shoveler, quail, red knot, redhead, ruddy turnstone, sanderling, scoter, semipalmated sandpiper, shorebird, turkey	486	
	2004–2017	H10N8	American green-winged teal, common scoter, environment, green-winged teal, gull (unspecified), laughing gull, long-tailed duck, mallard, northern shoveler, ruddy turnstone, shorebird	29	
	1996–2016	H10N9	Blue-winged teal, environment, northern shoveler, ruddy turnstone, shorebird	24	
Vietnam	2016	H10N6	Duck (unspecified)	1	1

a Only strains with a specified NA subtype are included.

## GENOMIC SEQUENCE AVAILABILITY AND NOMENCLATURE

### Availability of sequence data.

As of 15 June 2021, 1,367 full or majority partial (>70% of segment length) HA sequences were publicly available on FluDB (www.fludb.org). Most sequences are from North America (69%; *n* = 943), of which 94% were sampled in the United States. Additionally, 23% of sequences were sampled in Asia (*n* = 304), of which 79% (*n* = 239) were sampled in China. Approximately 8% of H10Nx sequences (*n* = 113) were sampled in Europe. Lower sequence availability outside the United States and China may partly reflect lower surveillance or reduced sequencing capacity in these regions (30). Increasing global sequencing in these regions for both H10 and other neglected subtypes is important for several reasons, not only to keep track of regional increase in occurrences in domestic poultry but also to identify potential reassortants that could allow cross-species and zoonotic transmission (31).

### Nomenclature.

While all AIVs have evolved into distinct lineages, which are typically characterized by geographical distribution, the rapid evolution and persistence of the H5 HA derived from the A/Goose/Guangdong/1996 (H5N1) strain incited the development of a standard clade nomenclature system. Phylogenetic analysis of HA genes characterized sequences into clades based upon sequence homology and specific clade definition criteria. The nomenclature system is relatively fluid; as viruses continue to evolve, new sublineages intermittently emerge until the specific clade definition criteria are met, and they are then assigned as separate clades (32). There is currently no universally recognized or formal nomenclature system for H10 viruses, and the clades that have been widely acknowledged are loosely characterized. Zhuang et al. (33) have proposed that H10 subtypes can be classified into two primary lineages, H10.1 and H10.2, which predominantly correspond with geographic location and AIVs circulating in the Western and Eastern hemispheres, H10.1 being North American and H10.2 referring to Eurasian viruses (33). Phylogenetic analysis of the H10 HA sequences available in the NCBI Influenza Virus Resource Database further confirmed that the H10 viruses can be divided into North American and Eurasian lineages (34). The Eurasian lineage is considered more divergent and has split into multiple sublineages, including European and Asian (JX436-like) (35).

## H10Nx VIRUSES IN AVIAN SPECIES

### Wild aquatic birds.

Wild birds are the natural host reservoirs of AIVs (36, 37). Avian influenza H10 viruses appear to be endemic in wild bird populations and have been detected globally in Canada, South Korea, Sweden, Italy, the United States, South Africa, and Japan since 1965 (38–42). More recently, H10 viruses have developed endemicity in Australian wild aquatic birds and are derived from several viruses circulating in waterfowl along various flyways (43). Their HA gene was derived from aquatic birds in the United States, whereas the NA gene is closely related to that from viruses previously detected in Japan. The remaining genes were derived from Eurasian avian influenza virus lineages. The low pathogenicity of the H10 viruses in birds makes them difficult to detect, allowing the virus to persist and spread; thus, wild birds and poultry can act as large reservoirs for virus maintenance in nature (44, 45).

### Domestic poultry.

**(i) H10Nx infections in domestic poultry in Asia.** *(a) China*. Active surveillance of AIVs has been ongoing in China for decades in an attempt to control outbreaks in poultry and humans, predominantly driven by live-bird markets (LBMs). Surveillance from 2002 regularly identified H10 in wild and domestic ducks, but this type was not identified in terrestrial poultry until 2013 (20). Since then, H10Nx viruses of multiple subtypes have been detected in domestic and terrestrial poultry with increasing frequency. Three H10Nx subtypes—H10N3, H10N7, and H10N8—were isolated from chickens in Zhejiang Province in eastern China during surveillance of AIVs in live poultry markets in 2016 and 2017 (46). Phylogenetic analysis indicated that these Chinese-isolated viruses received gene segments from H10, H3, and H7 viruses from birds in East Asia.

*(b) Bangladesh*. H10 AIVs have been frequently isolated with NA subtypes (N1, N6, N7, and N9) in LBMs in Bangladesh (47). Genetic analyses demonstrated that there are two antigenically distinct groups of H10 AIVs are circulating in Bangladeshi LBMs.

**(ii) H10Nx infections in domestic poultry in Australia.** H10N7 viruses detected in domestic poultry and abattoir workers in Australia in 2010 were found to have an HA gene of North American origin (43). The genomic origins indicated that the H10N7 viruses isolated from poultry were similar to those that have been circulating since 2009 in Australian aquatic birds and that their initial transmission into Australia occurred between 2007 and 2008. While H10Nx viruses have developed endemicity in Australian wild birds, there have been no recent incursions into domestic poultry (43).

## H10Nx VIRUSES IN MAMMALIAN SPECIES AND ZOONOTIC INFECTION

### H10Nx infections in humans.

In recent years, avian-origin H10 AIVs have proved capable of infecting humans and subsequently pose a potential public health threat (46).

In 2004, H10N7 influenza virus was detected in Egypt (48). Since this detection, the H10N7 AIVs have also caused sporadic human infections with variable clinical symptoms globally. An H10N7 subtype influenza virus (A/Sydney/2/2010) caused infection of poultry abattoir workers in Sydney in 2010 (49). Despite the sporadic incursion of H10N7 human infections, there is limited information pertaining to the molecular characteristics of H10N7 viruses, especially in China (50). An in-depth phylogenetic analysis conducted on chicken isolates by Wu et al. (50) indicated that the viruses contained genetic material from H10, H2, H7, and H3 AIV strains that were circulating at the same time.

Infection with a novel H10N8 influenza virus in humans was first described in China in December 2013. H10 is an LPAIV in avian hosts, allowing undetected circulation and the development of reservoirs (45, 51). An additional risk factor is the frequency of H10Nx reassortment (21, 46). H10Nx viruses have reassorted with enzootic AIV strains, which subsequently caused human infection; an example is that chicken H10N8 viruses identified in China were generated through multiple reassortments between H10 and N8 viruses from domestic ducks and the enzootic chicken H9N2 viruses. H9N2 is an LPAIV; however, it is endemic to much of Asia and the Middle East. There have already been several cases of H9N2 viruses breaching the zoonotic species barrier to infect humans, with this subtype being considered a human pandemic risk (52, 53). H9N2 viruses are also a source of much reassortment within bird populations, with transfer of a H9N2 internal gene cassette being common (54, 55). H7N9 is thought to use H9N2 as a source of reassortment when infected chickens are in close proximity and has subsequently caused a large number of human infections and deaths (55). These H10 chicken reassortant viruses were highly similar to the H10N8 human isolate identified in 2013, indicating that market chickens were the source of this human infection. This novel reassortant virus was confirmed to have originated from an LBM. The first fatal case of H10N8 was identified in a 73-year-old immunocompromised woman in China in 2014. This strain, named JX346, was established as a reassortant with PB1, PB2, PA, NP, M, and NS genes from H9N2. The PB2 gene also harbored mammalian adaptations for enhanced replication (56). The continued reassortment using the H9N2 internal gene cassette suggests that it may be a genetic platform to aid avian strains to cross the species barrier into humans (57).

More recently, the H10 viruses further reassorted, apparently with H5N6 viruses, and generated an H10N6 variant. A recent 2021 reassortant of H10 with N3 subtypes, A/Jiangsu/428/2021 (JX428), also caused human infection in China. Interestingly, the patient had no clear history of exposure to poultry prior to illness onset, based on epidemiological investigation. No avian H10N3 virus has been found in the local surroundings or poultry. Phylogenetic analysis of the novel JX428 isolate revealed it to be a reassortant between H10N3 and H9N2 viruses circulating in 2017 to 2019. HA and PB2 proteins both contain avian adaptation markers, such as 226Q and 627E, respectively. However, PB1 and M1 have adaptation markers linked to increased replication in mammalian cells and increased pathogenicity in mice. This novel H10N3 virus appears to have dual human and avian receptor preference but is unlikely to be transmitted between humans (8).

### H10Nx infections in swine.

While wild birds are the natural host reservoir for IAVs, swine are susceptible to infection with both avian and human AIVs. Swine are therefore regarded as the “mixing vessel” for influenza virus reassortants (58). H10N8 specific influenza virus antibodies were detected in swine herds in southern China during routine serological monitoring for swine influenza virus, following the detection of the same virus in humans in 2013. The pathogenicity and transmissibility of this H10N8 influenza virus to swine were examined and demonstrated that swine are susceptible to infection with human-origin H10N8 influenza virus, which causes viral shedding, severe tissue lesions, and seroconversion, while infection with avian-origin H10N8 influenza virus causes only seroconversion and no viral shedding (59). However, human-origin H10N8 influenza virus has inefficient transmission between swine and causes seroconversion only through direct contact (59). An avian-origin H10N5 influenza virus, A/Swine/Hubei/10/2008 (H10N5), was also isolated from pigs in Hubei, China (60). Phylogenetic analysis showed that the strain was wholly of avian origin and closely homologous to the Eurasian/H10.2 lineage viruses. This is regarded as the first report of interspecies transmission of an avian H10N5 influenza virus to domestic pigs under natural conditions (60).

The increasing prevalence of H10Nx viruses in swine highlights the importance of epidemiological monitoring of these H10 IAVs in different animal species, which is crucial in preventing and controlling future H10Nx infections.

### H10Nx infections in other mammalian species.

**(i) H10Nx infections in mink.** The first H10 mammal influenza virus was reported to have been isolated from mink in Sweden in 1984 (61, 62). Six strains of an IAV were isolated from a mink farm, with all six isolates being of the H10 subtype in combination with N4 (62). The unique property of some avian H10 viruses, particularly the ability to cause severe disease in mink without prior adaptation, is of great interest (63). Zohari et al. (48) investigated the influence of different genes on H10 AIV virulence in mink and genomic relationships between H10Nx strains. The genomic relationship between both the mink isolate (A/Mink/Sweden/3900/84) and other H10N4 viruses circulating in avian species at the same time, A/Fowl/Hampshire/378/85 and A/Mallard/Gloucestershire/374/85, showed high similarity between strains. A historic sample was also analyzed, A/Chicken/Germany/N/49, i.e., the H10 prototype virus (H10N7) (64), which demonstrated a weaker genomic relationship, possibly attributed to both virus evolution and reassortment (63). An additional strain, A/Whistling swan/Shimane/468/88 (H10N4), was also included. The H10 isolates presented in this study contained the amino acid sequence PQG|RGLF at the cleavage site in the HA molecule, indicating their low-pathogenicity genotype (48). However, four of these H10 viruses were found to be highly pathogenic in mink. The previously identified H10 isolates A/Turkey/England/384/7 (H10N4) and A/Mandarin duck/Singapore/8058F-72/7/93 (H10N5) (65) fulfil the criteria for high pathogenicity with an IVPI score of >1.2, despite not containing a multibasic cleavage site (65). This suggests that there are factors separate from the presence of multiple basic amino acids in the cleavage site contributing to the severity of H10 viruses in mink.

**(ii) H10Nx infections in dogs.** H10N8 viruses have also been reported to infect feral dogs in Guangdong Province, China (66). Canine sera collected in March to June 2013 from dogs living near LBMs were found to be reactive to JX346, approximately 6 months before the virus caused the death of a 73-year-old woman. Feral dogs have long-term exposure to birds and poultry entrails at LBMs, which increases their infection risk. Feral dogs may be able to act as sentinels for mammal-adapted viruses before human infection occurs (66).

**(iii) H10Nx infections in raccoons.** Raccoons have been found to be susceptible to multiple influenza subtypes, including H10, and to seroconvert (67). Raccoons have both α2,3- and α2,6-linked sialic acid cell receptor composition within the respiratory tract and so are susceptible to both human and avian viruses. Raccoons were shown to have H10 antibodies in the United States in 2004 and 2006 (68). Hall et al. (68) also showed that under laboratory conditions, raccoons were able to become infected, to shed virus, and to transmit it to uninoculated animals (68). Due to raccoons’ high mobility and the possibility of coinfection with both human and avian viruses, they are regarded as a possible mixing vessel host that could result in novel reassortant viruses (68).

**(iv) H10Nx infections in seals.** While H10Nx viruses have been recovered from numerous avian species, there has been sporadic transmission of these H10 viruses into mammals, including seals. Gull-origin H10N7 viruses were isolated in Iceland in 2015, and genomic analysis of these viruses demonstrated that four gene segments in the viruses were genetically associated with H10 IAVs that caused influenza outbreaks and deaths among European seals in 2014 (33, 69, 70).

### H10Nx infections in laboratory animals.

Mice are a convenient and relatively inexpensive model in which to study of the mechanisms of influenza virus pathogenesis and human polymerase adaptation. The virulence of four representative H10 AIV strains detected in Bangladeshi LBMs (one from each detected NA subtype) was assessed, and it was found that multiple H10Nx subtype viruses are able to replicate efficiently in mice without prior adaptation (47). Moreover, H10N6 and H10N1 AIVs caused high mortality with systemic dissemination (47). Wu et al. (50) conducted *in vivo* studies in mice with reassortant H10N7 viruses isolated from poultry in China. These viruses were found to be moderately pathogenic in mice. The viruses were also able to replicate in mice without prior adaptation (50). Mouse inoculation tests conducted by Wu et al. (46) also showed that three H10Nx subtypes—H10N3, H10N7, and H10N8—isolated from chickens in eastern China have low pathogenicity in mice and can efficiently replicate in this species (46).

A study by Lambertz et al. (71) found that H10 pathogenesis in mice is reliant on the presence of TMPRSS2 (transmembrane protease serine 2). TMPRSS2 has been shown to be responsible for the pathogenesis and cleavage of H1 (72) and H7 (73) subtype AIVs *in vivo* and is key in cleavage of coronavirus spike proteins and subsequent pathogenesis, specifically of severe acute respiratory syndrome coronavirus 2 (SARS-CoV-2) (74). Mice deficient in TMPRSS2 showed no weight loss, no viral replication, and a large reduction in inflammatory lesions within the lung compared to wild-type mice when infected with a H10 HA reassortant virus.

Ferrets are a widely used animal model for human influenza transmission and research, justified by the distribution of sialic acid receptors in the upper respiratory tract, which is similar to that in humans. They also exhibit comparable clinical signs when infected with influenza viruses (75–78). Transmission studies in ferrets with gull-origin H10N7 AIVs isolated from Iceland in 2015 demonstrated that the gull-origin viruses could infect ferrets (33) and that the virus could be transmitted between ferrets through both aerosol droplets and direct contact.

These studies demonstrate that H10Nx infections can infect mammals and effectively be transmitted among them without prior adaptation. H10Nx viruses are therefore a candidate for influenza pandemic preparedness and should be monitored in animal species as well as at the animal-human interface (33).

## ADAPTATION AND MUTATIONS IN H10Nx VIRUSES

### Poultry adaptation.

It is thought that poultry adaptation of IAVs can enhance the potential of an avian virus to cross the species barrier from avian to human (79). A major difference between viruses of terrestrial poultry origin and ancestral duck isolates is their different receptor binding profiles. AIVs are often transmitted from wild waterfowl to domestic poultry through the fecal-oral route (80); however, viruses circulating in different avian species differ in their fine receptor binding specificity and must adapt when switching, for example, from ducks to chickens (81). AIVs can also infect more than 105 bird species, allowing a deep reservoir for spillover events with an abundance of different glycan binding profiles (82). The glycan sugar repertoire in terrestrial poultry differs significantly from that in aquatic birds. Both α2,6- and α2,3-linked sialic acid receptors are present in chickens, specifically in the nasal cavity, upper respiratory tract, and gut, whereas in ducks, α2,3-linked sialic acid outweighs the proportion of α2,6 receptors (83–87). During poultry adaptation, the HA evolves; this may induce increased fitness for human receptors (78) but could equally adversely affect the ability of the HA to support virus transmission in humans (30).

A phylogenetic trace of the history of H10N8 viruses suggests that the virus spilled over from wild birds to poultry in southern China (34). An updated analysis showed that H10 HA sequences have not significantly diversified in recent years; however, the emergence of new lineages of internal genes and the global predominance of H9N2 in circulation should be noted due to consistent reassortment of H10Nx viruses (33). Viruses isolated from LBM in Zhejiang in 2016 to 2017 showed evidence of reassortment between H10, H3, and H7 viruses of Eurasian lineages (46). Characterization of H10N8 viruses isolated from Dongting Lake in China showed that polymerase genes were acquired by reassortment with H5 and H7 viruses circulating in Eurasian waterfowl (88). This situation increases the potential for the virus to develop mutations and undergo reassortment, endowing it with high pathogenicity and improved transmissibility to humans (89, 90). These findings also suggest that H10 AIVs have the ability to adapt extremely quickly to terrestrial poultry and highlight the need for long-term surveillance.

### Mammalian adaptation.

Upon transmission to a new host, the virus must successfully bind to, enter, and replicate in the host cell as well as evading the innate immune response long enough for egression and then transmission to a new host (30). Consequently, there are a number of factors, all of which are essential, in adaptation to new host species. Certain avian H10Nx viruses have been shown to successfully infect mammalian hosts with minimal or no host adaptation required (45, 46, 91) (Table 2).

**TABLE 2:** Mammalian adaptation mutations in H10Nx viruses (H3 numbering)

Protein	Strain	Subtype	Species	Infection type	Mutation(s)	Effect	Reference
HA	A/Seal/Sweden/SVA0546/2014	H10N7	Seal	Natural	351H, 379I, 398D	Mammalian adaptation in seals	102
	A/Jiangsu/428/2021	H10N3	Human	Natural	Q226L, E627K	Allows binding to both human- and avian-like receptors	8
	A/Mallard/Beijing/27/2011 (MA; BJ27-MA)	H10N7	Mouse	Laboratory	G218E	Affects both the receptor specificity and the pH of fusion	91
	A/Jiangxi-Donghu/346/2013	H10N8	Human	Natural	A135T, K137R, S138A	Mammalian adaptation and increased virulence in HA	45
					Q226, S227, G228S	Change the binding preference from the α2,3 avian receptor to the α2,6 human receptor.	
					E190	Allows binding to both human- and avian-like receptors	
	A/Harbour seal/Netherlands/PV14-221_TS/2015	H10N7	Seal	Natural	Q226L, T224I, E74D	Mammalian adaptation in seals (Q226L); HA stabilization (T224I and E74D)	102
PB2	A/Mallard/Beijing/27/2011 (MA; BJ27-MA)	H10N7	Mouse	Laboratory	E158G	Mediates increased virus replication and severity of infection in mice and mammalian cells.	91
					M631L	Strongly upregulates viral polymerase activity and virus replication and severity in mice and mammalian cells	
	A/Seal/Sweden/SVA0546/2014	H10N7	Seal	Natural	17C, 453S	Mammalian adaptation in seals	102
	A/Environment/Dongting Lake/Hunan/3-9/2007	H10N8	Environment	Natural	E627K	Enhanced polymerase activity	88
	A/Jiangxi-Donghu/346/2013	H10N8	Human	Natural	E627K	Enhanced replication in ferrets and mammalian adaptation	114
	A/Duck/Fujian/1761/2010	H10N3	Mouse	Laboratory	E627K, Q591K	Mammalian adaptation and increased virulence in BALB/c mice	35
PB1	A/Seal/Sweden/SVA0546/2014	H10N7	Seal	Natural	752D	Mammalian adaptation in seals	102
	A/Jiangsu/428/2021	H10N3	Human	Natural	V473D	Enhanced polymerase activity and increased replication in mammalian cells	8
PA	A/Seal/Sweden/SVA0546/2014	H10N7	Seal	Natural	192H	Mammalian adaptation in seals	102
M1	A/Jiangxi-Donghu/346/2013	H10N8	Human	Natural	N30D, T215A	When occurring concurrently, increased virulence (as seen in H5N1)	114
	A/Jiangsu/428/2021	H10N3	Human	Natural	31N, 215A	Increased pathogenicity in mice	8
M2	A/Jiangsu/428/2021	H10N3	Human	Natural	31S	Increased amantadine resistance	8
NS1	A/Jiangxi-Donghu/346/2013	H10N8	Human	Natural	P42S	Increased pathogenicity in mice	114
NA	A/Harbour seal/Netherlands/PV14-221_TS/2015	H10N7	Seal	Natural	247I, 436T	Mammalian adaptation in seals	102
	A/Mallard/Beijing/27/2011 (MA; BJ27-MA)	H10N7	Mouse	Laboratory	K110E, S453I	Significantly promotes NA enzymatic activity and mammalian adaptation	91

### HA structure, HA mutations, and receptor preference.

IAV host adaption is partially dependent on the binding of the viral HA to specific sialic acid isoforms. Avian-like viruses favorably bind sulfated or nonsulfated α2,3 sialic acid isoforms, whereas human influenza viruses preferentially bind the α2,6 isoform (92–94). The receptor-binding specificity of IAVs is also a major determinant of virus-host tropism, which allows interspecies transmission (30). H10Nx viruses have different propensities to bind α2,3 or α2,6 sialic acids, depending on lineage and virus origin.

H10 HA crystal structures of human-origin JX34 (H10N8) and avian-like H10N2 showed that H10 is structurally diverse compared to other HA subtypes (95). The residues that are known to modulate receptor preference in other HA subtypes are conserved in the receptor binding site (RBS) of JX34 and avian-like H10N2 HA (95). The JX34 H10HA complex with the human α2,6 isoform displays a 45° difference in the angle of rotation about the Gal-2 C6–C5 bond, which changes the RBS exit trajectory of the human analogue to a less vertical fashion than that seen in pandemic H1 and H3 HA receptor complexes. The human analogues also exit to the side of the H10HA RBS in a trajectory similar to that of a human H7HA-α2,6 isoform cocomplex. This receptor configuration has been correlated with the presence of L226 mutations, commonly seen in human in H7 viruses. However, the H10HA has a Q226 residue, not usually associated with mammalian adaptation. Wang et al. reported that the structure of the JX346 H10HA also bound to avian receptor analogues (45, 60, 95–97). The HA RBS of JX346 displays not only the signature Q-S-G residues at positions 226 to 228 (H3 numbering) but also E190, which is indicative of an avian-like HA RBS (98). Additionally, when this virus was serially passaged in embryonated hens’ eggs, no mutations arose. However, when serially passaged in mammalian cell culture (Madin-Darby canine kidney [MDCK] cells), it produced eight point mutations, enforcing the theory of high efficiency for adaptation to avian hosts (99).

Glycan binding preference analysis conducted on gull-origin H10N7 viruses isolated in Iceland in 2015 suggest that, similar to other avian-origin H10 IAVs, the viruses bound to both avian-like α2,3-linked sialic acids and human-like α2,6-linked sialic acids (33). However, when the gull-origin H10N7 viruses were compared to JX346, which caused human infections, the gull-origin viruses showed significantly higher binding affinity to human-like glycan receptors, despite maintaining an avian host (33). Additionally, despite human infection, both JX346 and avian A/Green-winged teal/Texas/Y171/2006 were found to maintain α2,3 binding. As JX346 was not transmitted between human hosts, receptor preference appears to have been the limiting factor (45, 100).

Zhang et al. (100) have identified several key mutations responsible for increased affinity of human and avian-like receptors, including Q226L and/or G228S mutations in wild-type H10Nx viruses. They result in the loss of binding to α2,3 sialosides but do not increase fitness for binding to human-type α2,6 receptors (100).The JX346 virus presented two key residues within the HA which are known as genetic hallmarks for mammalian adaptation and virulence, A135T and S138A (98, 101). H10 has yet to be identified with a multibasic cleavage site, a characteristic of HPAIV, despite some strains exhibiting high pathogenicity in poultry (12).

During serial passage experiments in mice, mutations have arisen in the HA in conjunction with PB2 and NA genes (91). An avian H10N7 outbreak in seals showed mutations in HA to alter receptor binding preference and stability, including the adaptation marker Q226L. In seals, although mutations arose in polymerase subunits, no difference in replication kinetics was found (102). Several seal isolates also had additional point mutations in the HA (351H, 379I, and 398D), contributing to increased fitness for mammalian adaptation.

With the increasing occurrence of H10Nx incursions into mammalian species, HA mutations should be closely monitored, along with continued analysis of the effect these HA mutations have with regard to receptor binding specificity. With the HA protein being responsible for receptor binding and viral entry and, therefore, receptor preference being a major determinant of both host tropism and zoonotic transmission, this is an area that cannot afford to be neglected.

### Recent developments.

Phylogenetic analysis of the novel H10N3 A/Jiangsu/428/2021 human isolate (JX428) revealed it to be a reassortant between avian H10N3 and H9N2 viruses circulating in 2017 to 2019. HA and PB2 proteins both contain avian adaptation markers such as 226Q and 627E, respectively, suggesting that it has dual binding specificity of both avian-like and human-like receptors (8). Despite this, this novel H10N3 virus appears to be unlikely to be transmitted between humans (8). Both PB1 and M1 have adaptation markers linked to increased replication in mammalian cells and increased pathogenicity in mice. The PB1 gene contains a 473D point mutation, which has been found to enhance polymerase activity of avian polymerase in mammalian cells (103). It also encodes a full-length PB1-F2 (8), which is associated with avian viruses, as avian progenitors lose the PB1-F2 open reading frame (ORF) through circulation and adaptation to the mammalian host (104, 105). Loss of PB1-F2 reduces virulence in mammals, leading to the hypothesis that the protein acquires truncations in order to prevent an intense host immune response (106). The JX428 NS1 does not contain a PDZ domain (8). Lack of a PDZ domain is highly unusual (107) and likely to affect virulence (108). The M2 protein contains the amantadine resistance marker 31S (8), which has worrying connotations if a zoonotic H10 were to acquire the ability to be transmitted between human hosts (8).

This novel human H10N3 virus is an avian-origin reassortant strain, with the HA and NA genes from H10N3 viruses and six internal genes from H9N2 poultry viruses, which possess interspecies transmission capability. These pose a very real threat to human health and have pandemic potential; therefore, it is essential to monitor the evolution of H10N3 viruses in Chinese LBMs and domestic birds to provide timely prevention and disease mitigation strategies for these influenza viruses (8).

## VACCINATION AND CONTROL

Currently, there are no approved vaccines against H10 AIVs; however, the frequent outbreaks within poultry populations and sporadic introductions, often fatal, into humans have heightened the urgency of precautionary vaccine developments. Investigations into the potential of virus-like particles (VLPs) (109), anti-HA head and anti-HA stalk monoclonal antibodies (110), and the traditional method of inactivated virus vaccine of the outbreak strain (111) have all shown promising protection against the potential lethality of infection *in vivo*. Additionally, at present, an mRNA vaccine targeting the HA of H10N8 (A/Jiangxi-Donghu/346/2013) has shown promising results following a phase 1 randomized double-blind clinical trial (112).

## CONCLUSIONS AND PERSPECTIVES

In recent years, the frequency of detection for H10Nx infections is increasing exponentially. Due to the high reassortant rate between H10 and numerous NA subtypes coupled with the nonnotifiable status of the virus, vast geographical distribution, and lack of sequencing outside Asia and North America, the true extent of H10Nx prevalence could be far higher than currently predicted.

With the increase in human cases, as well as noncontact infections of novel viruses, H10Nx infections continue to pose a threat to both human and animal health. H10 viruses also have endemicity in several regions, sitting across major migratory flyways, meaning that there is a very real threat of increased spread of novel H10Nx infections to both wild birds and domestic poultry and therefore other mammalian species.

Multiple studies have consistently highlighted the ease with which H10Nx viruses are able to reassort with circulating strains (35, 45, 46). Frequent reassortment, coupled with the minimal host adaptation requirements for human infection, clearly identifies H10Nx as posing a pandemic risk. This subtype needs to be subjected to higher levels of surveillance. H10N8 has crossed the species barrier from avian species into both swine and humans, suggesting that this is currently the most effective HA-NA combination for mammalian adaptation. Swine are susceptible to infection with both human and avian influenza viruses, which may undergo reassortment to generate novel reassortant viruses, with swine therefore being considered “mixing vessels” (113). H10N8 has been shown to efficiently replicate in swine and has also caused human fatalities; therefore, N8 is a key subtype to observe closely.

While IAV incursions from avian species to mammalian hosts occur, they are fairly infrequent, with a number of key mutational markers usually present, which are well documented (23). Several H10Nx viruses have been found to alter their PB2 sequence on adaptation to mammalian hosts. E627K has previously been identified as important for mammalian adaptation in other major AIV subtypes (35). In FJ1761 (H10N3) and JX346 (H10N8), E627K resulted in increased virulence in BALB/c mice and enhanced replication in ferrets (35, 45, 114). However, for H10N8 3-9/07, the E627K mutation did not appear to be directly linked to enhanced pathogenesis in BALB/c mice but was instead associated with enhanced polymerase activity (88). Additionally, a serial passage experiment of an H10N7 LPAIV in mice did not produce any PB2 E627K mutations (91). This renders H10Nx viruses unique in their ability to cross the species barrier, as unlike in some other AIV subtypes, E627K appears to be of less importance to avian H10Nx viruses in mammals.

Analysis by Vijaykrishna et al. (43) indicates that the long-term evolutionary dynamics of avian influenza viruses may be determined by climatic changes. Therefore, environmental factors affecting the dynamics of avian influenza should be considered when surveillance and disease control strategies are introduced (43).

Antiviral drugs are widely used to restrict IAV replication in human infections (115, 116). Baloxavir and favipiravir have been shown to be highly effective against numerous IAV subtypes, including H10 (115, 117). The increased emergence of drug-resistant influenza virus strains highlights the need for new antiviral therapeutics to combat future pandemic outbreaks as well as continuing seasonal cycles of influenza (118). While antiviral resistance markers have not yet been identified in H10Nx viruses, this should be closely monitored with the increase in H10 mammalian infections.

Additionally, the need for a formal nomenclature system, with clear and concise lineages in place would allow for greater understanding of the pandemic potential and subsequent risks of emerging H10Nx infections as well as a greater understanding of the distribution, tropism, and infection capabilities of these viruses.
